# The CRISPR conundrum: evolve and maybe die, or survive and risk stagnation

**DOI:** 10.15698/mic2018.06.634

**Published:** 2018-05-16

**Authors:** Jesús García-Martínez, Rafael D. Maldonado, Noemí M. Guzmán, Francisco J. M. Mojica

**Affiliations:** 1 Departamento de Fisiología, Genética y Microbiología. Universidad de Alicante, Campus de San Vicente, 03690 San Vicente del Raspeig (Alicante), Spain.; 2 I.M.E.M. Ramón Margalef. Universidad de Alicante, Campus de San Vicente, 03690 San Vicente del Raspeig (Alicante), Spain.

**Keywords:** CRISPR, Cas, prokaryotic adaptive immunity, horizontal gene transfer, prokaryotic evolution

## Abstract

CRISPR-Cas represents a prokaryotic defense mechanism against invading genetic elements. Although there is a diversity of CRISPR-Cas systems, they all share similar, essential traits. In general, a CRISPR-Cas system consists of one or more groups of DNA repeats named CRISPR (Clustered Regularly Interspaced Short Palindromic Repeats), regularly separated by unique sequences referred to as spacers, and a set of functionally associated *cas* (CRISPR associated) genes typically located next to one of the repeat arrays. The origin of spacers is in many cases unknown but, when ascertained, they usually match foreign genetic molecules. The proteins encoded by some of the *cas* genes are in charge of the incorporation of new spacers upon entry of a genetic element. Other Cas proteins participate in generating CRISPR-spacer RNAs and perform the task of destroying nucleic acid molecules carrying sequences similar to the spacer. In this way, CRISPR-Cas provides protection against genetic intruders that could substantially affect the cell viability, thus acting as an adaptive immune system. However, this defensive action also hampers the acquisition of potentially beneficial, horizontally transferred genes, undermining evolution. Here we cover how the model bacterium *Escherichia coli* deals with CRISPR-Cas to tackle this major dilemma, evolution versus survival.

## INTRODUCTION

The prokaryotic world has been historically the main source of tools for genetic engineering and molecular biology in general. CRISPR-Cas is a recent example of how the study of prokaryotes has revolutionized life sciences. Besides becoming the most important tool for genomic editing to date 1, the discovery of this immune system has marked an important milestone in the history of Microbiology.

Cas proteins, CRISPR loci and CRISPR RNAs are the core functional parts of an adaptive and heritable resistance system against foreign DNA. They enable the cell to keep memory of infections by exogenous elements and fight against the invader. There is a significant diversity of genes associated with CRISPR, presumably reflecting the selective pressure viruses exert on the evolution of the system. Classification of CRISPR-Cas systems has been proven a challenging task 2,3,4, and new variants are emerging as sequencing data increases and functional studies on these systems are performed. Distinct CRISPR-Cas systems can coexist in a genome 4,5,6,7,8. Moreover, the number of CRISPR loci pertaining to the same type varies among organisms, and both the identity and number of spacers within each array greatly changes even among genomes of closely related strains 9.

In this paper, we present an overview of the CRISPR-Cas systems outlining their discovery, classification and functional role, and we discuss about the evolutionary importance of these systems in the model organism *Escherichia coli*. The chromosome of *E. coli* strains may harbor up to two CRISPR-Cas systems involving as much as two repeat arrays each 6. Equivalent arrays show a considerable intraspecific polymorphism in terms of spacer number and sequence. Fundamental knowledge about the CRISPR-Cas mechanism has been generated from the analysis of these two systems in *E. coli *10,11,12,13 and related Enterobacteriaceae 14.

## DISCOVERY OF CRISPR LOCI AS DNA-MEMORY STORES

The serendipitous finding by Nakata and collaborators in 1987 15 of five direct repeats next to the *iap* gene in *E. coli* was the first report of a CRISPR locus. Subsequently, in 1989 16 the Nakata’s team documented another array of repeats at approximately 20 kb from the first one. Soon after, Hermans *et al.* 17 found direct repeats in the unrelated, Gram-positive *Mycobacterium tuberculosis* complex, launching the use of the repeat loci for strain typing based on their particular spacer content 18. Archaea first CRISPRs were discovered in 1993 19, and the earliest functional studies on these sequences were performed in 1995 20. By the end of the 1990’s, similar direct repeats were found in other prokaryotes and denominations given to these sequences started to multiply: DR, direct repeats 17; TREPs, tandem repeats 20; SRSR, short regularly spaced repeats 21; DVR, direct variant repeats 22; LCTR, large clusters of tandem repeats 23; SPIDR, spacers interspersed direct repeats 24. To avoid confusion, an agreement was made on naming the repeated sequences as CRISPR 25. This acronym appeared published for the first time in 2002 26. By then, the biological relevance of these sequences was recognized, since they were distributed among many different, distantly related prokaryotes, representing a widespread family of repeats 21. However, even though protein coding genes commonly associated to CRISPR arrays were discovered 26, unraveling their function was still pending. These Cas proteins, some of them related to helicases or nucleases, could play a role on DNA metabolism or expression^26^.

Nevertheless, the definitive hint for the biological function of CRISPR-Cas came from the spacers rather than from the Cas or CRISPR units. In 2005, three independent studies found that some spacers matched sequences from transmissible genetic elements 27,28,29. Notably, a comprehensive survey of the literature published on viruses and plasmids carrying spacer homologs, pointed to a relationship between immunity to these carriers and the presence of the cognate spacer in a potential host 27. Therefore, it was suggested that the spacers represent a memory of past infections, and this information might be used to guide a defense mechanism. This fundamental breakthrough in the understanding of the CRISPR role in nature came hand in hand with the advent of increasing amounts of sequence data generated from viral, plasmid and complete genome sequences of prokaryotic strains which allowed researchers to cross-compare them. The existence of an adaptive, immunity-like system in Bacteria and Archaea was such an innovative idea that the three research groups undergone difficulties in publishing their results 30. Historical perspectives of the initial moments of this discovery have been published elsewhere 25,30,31,32,33,34 showing interesting insights into the way modern science works and how scientific discoveries are made.

In 2007, the function of CRISPR-Cas as a specific immune system was experimentally proven in *Streptococcus thermophilus *35: phage resistance was endowed after the incorporation of small fragments of the foreign genetic material as spacers into the CRISPR loci of the bacterium. Moreover, Cas proteins were shown to be involved in this immunity. One year later it was demonstrated that transcripts derived from CRISPR arrays in *E. coli* were processed by Cas proteins and that the resulting small RNAs (crRNAs) are necessary to achieve immunity 12.

## CRISPR-CAS MECHANISM

Despite the diversification of CRISPR-Cas systems and their wide distribution in distantly related bacteria and archaea 4, the fundamental mechanism of this immune system is quite conserved, following three basic steps: adaptation, expression and interference.

Adaptation, or spacer acquisition, requires the integration of fragments of nucleic acids from invader molecules 36,37,38. In addition to Cas, non-Cas proteins are involved in this stage 39. Fragments of foreign nucleic acids selected for integration, named proto-spacers 40, are usually flanked by short conserved sequences, the proto-spacer adjacent motif (PAM) 41. New spacers are preferentially integrated in a polarized manner 29, next to the terminal CRISPR unit downstream to an AT-rich region called leader 12,26,42. The PAM sequence is needed for most, but not all systems to recognize foreign targets, and its absence in the own CRISPR array avoids self-targeting 43. Most CRISPR-Cas systems acquire spacers directly from DNA donors but a few systems are able to gain new spacers derived from RNA precursors after retrotranscription 44.

The transcription of a CRISPR array from the leader generates a multi-spacer RNA (pre-crRNA) which is processed to single-spacer crRNAs with the participation of Cas proteins 12 and, in some systems, of non-Cas ribonucleases as well as a trans-activating crRNA (tracrRNA) that partially hybridizes with the pre-crRNA 45. After processing, each mature crRNA (or crRNA/tracrRNA duplex) remains assembled with Cas proteins in a CRISPR ribonucleoprotein (crRNP) complex 46,47. This completes the second step of the CRISPR mechanism.

During the interference stage, the crRNP complex recognizes and directs cleavage of spacer-complementary sequences resulting in the elimination of molecules that carry potential targets 48. Specific PAMs are crucial for efficient interference by many CRISPR-Cas systems 48,49,50. In this case, upon PAM recognition by a protein of the crRNP complex, double-strand pairing is disrupted at the target DNA, leading to a R-loop conformation through progressive hybridization (starting from the PAM) with the spacer sequence in the crRNA 46. The R-loop is the substrate for cleavage by Cas endonucleases 51. Some CRISPR-Cas systems target RNA instead of, or in addition to, DNA 52,53,54.

## CRISPR-Cas SYSTEMS CLASSIFICATION

Cas proteins are categorized in three functional modules 55. The suite of proteins for the acquisition module is quite uniform. Regular members are Cas1 and Cas2 36,56, which have nuclease activities and form a multi-protein complex 57. The Cas1-Cas2 adaptation complex appears to be assisted by Cas4 when present, and might be included in this module 58,59. In contrast to the acquisition proteins, the effector module (that is, proteins involved in pre-crRNA processing, target recognition and cleavage) is highly variable 3,4,60. There is a third module of ancillary Cas proteins, involved in regulatory and other unknown roles 3,60.

Due to the fast evolution and wide diversification of the CRISPR-Cas systems, a multiple criteria approach has been used for classification: signature *cas* genes specific for some types, sequence similarity between common Cas proteins, the phylogeny of Cas1 (the most conserved Cas protein) and gene configuration in the loci 3,4. The application of these criteria resulted in the current classification principle of two classes (1 and 2) and six types (from I to VI) 3. Several subtypes (designated by letters, from 'A' forward) have been proposed based on signature genes and characteristic genomic arrangements 3,4. Moreover, at least in the case of *E. coli*, subtype variants showing substantial differences in *cas* sequence and PAM preference have been recognized within the species 61,62. This classification system also involves a systematic naming for Cas proteins that, in some cases, has changed over time to adapt to new discoveries 2,3,4.

Class 1 systems rely on multi-protein effector complexes 3. They include Type I and Type III systems (distinguishable by the presence of Cas3 and Cas10, respectively) as well as the uncommon Type IV, devoid of an adaptation module. Class 2 is defined by the presence of a single-protein effector, namely Cas9, Cas12 or Cas13, depending on the particular type of system (Type II, Type V and Type VI, respectively) 3,63. In spite of the need for tracrRNAs by Type II systems 45, not being involved in Class 1 systems 3, most applications of CRISPR technology in heterologous hosts are based on Type II components. This is mainly because, in contrast to Class 1, a single protein is required for interference and the target is cleaved just once at precise sites 31.

## HOW PROKARYOTES BYPASS THE GENETIC BARRIER DICTATED BY CRISPR: THE CASE OF *Escherichia coli*

Once the biological function of CRISPR-Cas was revealed, the potential drawbacks that fully efficient CRISPR-mediated interference could pose to prokaryotic evolution became evident 64. Horizontal Gene Transfer (HGT) is one of the main forces driving genetic change in Bacteria and Archaea 65,66. However, the uptake of foreign nucleic acids might be constrained by functional CRISPR-Cas systems. To cope with such situation, prokaryotes either lack these systems or place them under stringent control 67,68. This is exemplified by the case of *E. coli*, a paradigm of genome plasticity 69,70 in spite of being in possession of CRISPR-Cas systems 6: a subtype I-E system is present in the majority of strains and a complete I-F system exists in a reduced number of isolates. Still, cells harboring *cas* genes of the two subtypes are extremely rare 6,61. Unexpectedly, the *E. coli* I-F system is constitutively expressed under normal laboratory growth conditions 71,72. Therefore, in principle, it is permanently acting against gene transfer. However, the PAMs of the spacers present in the I-F arrays of *E. coli* differ from the proto-spacer adjacent motifs that elicit the most efficient interference 71. Such a relaxed interference could provide the opportunity for beneficial foreign DNA to be acquired, while at the same time still limiting exchange of unwanted genetic material. Remarkably, when I-F *cas* are absent in the *E. coli* genome, an array with a limited number of I-F repeats is invariably present, allegedly as a remnant of an ancient complete I-F system 6,61,73. Most strikingly, the vast majority of spacers in these orphan arrays match *cas* I-F genes 6,73, playing a crucial role in preventing the barrier effect of their cognate genes 73. Strains harboring these arrays use them as a constitutively expressed anti-*cas* mechanism that avoids the establishment of a fully equipped, immunity-prone CRISPR-Cas I-F system: intrusive DNA containing *cas* I-F genes is degraded through the action of the encoded Cas proteins guided by the resident crRNAs 73. This anti-*cas* mechanism strongly supports the hypothesis that CRISPR-Cas immunity could be annoying for the carrier cell.

Opposite to I-F, expression of the *E. coli* I-E system is precisely regulated. H-NS protein is the main repressor of the system and its silencing effect can be lessened by the transcription factor LeuO 74,75,76. The cAMP receptor protein (CRP) also contributes to CRISPR inhibition, acting as a competitor of LeuO for binding to the regulatory regions in the CRISPR-*cas* locus 77. However, activity of the I-E system of *E. coli* has not been detected under the multiple laboratory growth conditions so far tested (our unpublished results), and the natural circumstances upon which such silencing is relieved remain to be clearly elucidated. In this regard, quorum sensing autoinducers of the N-Acyl-homoserine-lactone (AHL) class appear to activate CRISPR-Cas systems in Gram-negative bacteria such as *Pseudomonas aeruginosa* 78 and *Serratia* sp. 79 at elevated cell densities, when the risk of infection by bacteriophages is the highest 80. Although this sort of induction has not been detected in pure cultures of *E. coli*, the presence in this species of AHL receptors 81,82 raises the possibility that their CRISPR-Cas systems might be regulated through an interspecific crosstalk, by signals secreted by other members of the microbial community. Overall, these findings illustrate the complexity of I-E CRISPR-Cas regulation in *E. coli*. Moreover, its diverging spacer count and identity within the species is an indication that CRISPR activity, at least at the adaptation stage, is turned on at a different pace depending on the particular group of strains.

Related to this, a notable case is that of pathogenic strains. When compared to non-pathogens (i.e., commensals), they gain a selective advantage via the acquisition through HGT of virulence factors, allowing them the ability to colonize more varied ecological niches within their hosts 70,83,84. Inquiringly, a recent work from our group 85 established a negative correlation between pathogenicity and I-E CRISPR repeat count in *E. coli*: commensal strains tend to have more repeats than pathogenic isolates. This observation is compatible with the hypothesis that the activity of CRISPR-Cas I-E is kept limited when environmental adaptation needs to take precedence over protection, to minimize the negative effects of an evolutionary constraint. Another related question is why *E. coli *strains have lost either the I-E or the I-F *cas* genes, depending on their particular environment 6,85. Indeed, most extra-intestinal pathogens pertaining to diverse phylogroups retain a I-F CRISPR-Cas system while the majority of commensals and enteric pathogens harbor a I-E system 85. The preference for one or the other CRISPR-Cas subtype is suggestive of functional differences between the two systems. In this sense, previous works have reported that whereas spacers within I-E arrays of *E. coli* target viruses and plasmids alike, most I-F spacers matching known sequences have a plasmid origin 6,72,85. Being plasmids the primary vectors for antibiotic resistance genes 86, this bias of I-F toward targeting plasmids is in agreement with the observation that the carrier strains are particularly susceptible to antibiotics 72. Even though the reason for this apparent specialization is unknown, it highlights the inconvenience of an indiscriminate interference and the burden of carrying multiple CRISPR-Cas systems.

In summary, the analysis of the different CRISPR-Cas settings found in *E. coli *strengthen the idea that these systems, despite conferring protection, could severely hamper prokaryote evolution, hinting at how detrimental they could become if left unrestricted. Therefore, avoiding *cas* genes and limiting CRISPR-Cas activity when present appears to be a necessary evil for a prokaryote, where a delicate balance should be reached between the two extremes, those of promiscuity or chastity in terms of genetic exchange.
